# Covid‐19: Supporting nurses' psychological and mental health

**DOI:** 10.1111/jocn.15307

**Published:** 2020-06-02

**Authors:** Jill Maben 1,2,3,✉, Jackie Bridges

**Affiliations:** ^1^ Professor of Health Services Research and Nursing Faculty of Health and Medical Sciences School of Health Sciences University of Surrey Guildford UK; ^2^ Faculty of Health University of Technology NSW Sydney Australia; ^3^ Discipline of Nursing, College of Science, Health, Engineering and Education (SHEE) Murdoch University Perth WA Australia; ^4^ Professor of Older People's Care School of Health Sciences University of Southampton Southampton UK; ^5^ NIHR Applied Research Collaboration Wessex Innovation Centre Southampton Science Park Chilworth, Southampton UK

At the time of writing (11 April 2020), there are 1.72 million COVID‐19 infections and 104,889 deaths worldwide. In the UK, the first recorded death was on the 5 March 2020, and in just 37 days, 9,875 deaths in hospital have been recorded. The 10 April saw the highest number of UK daily deaths (980) to date. These UK figures do not include those who died in care homes or in the community. Similar death rates have been experienced in China earlier this year (3,339) and are rising globally with particularly high death rates in the USA (18,761 with over half of deaths in New York State), Italy (18,939), Spain (16,353) and France (13,197). The UK death rate now stands at 29,427 including care homes (5th May 2020), the highest in Europe.

"I broke down and cried today. I cried of exhaustion, of defeat. Because after 4 years of being an ER nurse, I suddenly feel like I know nothing" (Sydni Lane, USA, Instagram and Facebook). (Fick , 2020)

“It's an experience I would compare to a world war” Roberta Re, Italy. (Giuffrida, 2020)

“we're on our knees here, and it's really difficult and we're all trying the best we can and we don't feel… we feel like we could be doing more, and I know we can't … we're staying away from our families and we're putting ourselves in danger to try and save other people's loved ones, it feels like a losing battle but it's not, we've all got hope and we're all trying to do what we can.” (Shirley Watts, UK ICU Nurse, BBC news 04 April 2020)

As the coronavirus disease 2019 (COVID‐19) pandemic takes hold, nurses are on the front line of health and social care in the most extreme of circumstances. We reflect during a moment in time (week three of lockdown in the UK and week 5/6 across Europe) to highlight the issues facing nurses at this unprecedented time.

At the bedside 24 hr a day seven days a week, in similar outbreaks, nurses have had the highest levels of occupational stress and resulting distress compared with other groups (Cheong & Lee, 2004; Maunder et al., 2006; Nickell et al., 2004). Nurses are already a high‐risk group, with the suicide rate among nurses 23% higher than the national average (ONS, 2017). Despite this, the RCN (Royal College of Nursing in the UK) has reported that nurses feel “repeatedly” ignored by their employers when they raise concerns about their mental health (Mitchell, 2019). A focus on personal responsibility for psychological health and well‐being and an overemphasis on nurses being “resilient” in the face of under‐staffing and often intense emotional work is consistently challenged by nurses and nurse academics (Traynor, 2018). Treating resilience as an individual trait is seen to “let organisations off the hook” (Traynor, 2018), yet has often been the focus of organisational strategies to date. This does not work at the best of times and certainly is not appropriate now in these most difficult of circumstances.

Here, we discuss the stressors and challenges and present evidence‐informed guidance to address the physical and psychological needs of nurses during the COVID‐19 pandemic. We stress the importance of peer and team support to enable positive recovery after acutely stressful and emotionally draining experiences, and outline what managers, organisations and leaders can do to support nurses at this most critical of times.

## 1 COVID‐19 STRESSORS AND CHALLENGES

The high prevalence of COVID‐19 in the general population of many countries, its novelty and highly infectious nature, and the associated morbidity and mortality rates are placing an unprecedented demand on health and social care services worldwide. In addition to the admission to hospital of high numbers of critically ill patients, care demands on nurses and care assistants have also increased in the community, in care homes and in learning disability and mental health services. These demands must be met by an already‐depleted workforce (+44,000 RN vacancies in the UK pre‐COVID‐19) and one that is further depleted at this time due to infection, self‐isolation and family responsibilities in the face of the crisis.

The nature of care itself and new ways of working are potentially highly stressful for staff. Nurses are not only experiencing an increase in the volume and intensity of their work, but are having to accommodate new protocols and a very “new normal.” For instance, many mental health services have transformed almost overnight from providing face‐to‐face care and treatment to a predominately virtual service of telephone or video consultations. In many other areas, nurses are adjusting to providing end‐of‐life care more frequently and often in the face of more rapid deterioration than they are used to. Isolation rules mean the presence of family at the bedside is rarely possible. Nurses are therefore frequently standing in for family members and facilitating remote access for loved ones.

Established nurse–patient ratios are under strain. In ITU in the UK, for instance, staff‐patient ratios of one‐to‐one are changing to ratios of one ITU nurse to six or more patients, with the shortfall being made up by staff without ITU experience. To boost the nursing workforce, many countries have also fast‐tracked their final‐year nursing students to join the nursing register early and have encouraged retired colleagues back to practice (Jackson et al., 2020). Many nurses have been redeployed, working in new specialities or in higher acuity areas. All of these factors are likely to be adding stress for existing staff, with additional implications for the well‐being of new members of the team.

Evidence from studies on COVID‐19 and other infectious respiratory disease outbreaks reflects high concern among nurses for personal or family health in the face of direct contact with a potentially deadly virus and the stress of balancing this concern with the ethical obligations of continuing to provide care (Jiang, 2020; Khalid, Khalid, Qabajah, Barnard, & Qushmaq, 2016; Kim & Choi, 2016; Nickell et al., 2004). Other stressors evident from research to date include concerns about shortages of staff and of personal protective equipment (PPE), navigating an unfamiliar setting or system of care and lack of organisational support (Kim, 2018; O'Boyle, Robertson, & Secor‐Turner, 2006; Shih et al., 2009). Additionally psychological conflicts between healthcare workers' responsibility to care for the ill and their right to protect themselves from a potentially lethal virus were reported (Chen, Wu, Yang, & Yen, 2005).

Our own anecdotal sources in the UK and Europe endorse these findings for COVID‐19 as (at time of writing) we approach the peak of the pandemic, but also raise the possibility of other stressors including moral distress resulting from treatment decisions based on finite resources, the lack of access to antigen or antibody testing for most front‐line staff, and the discomfort and fatigue resulting from long shifts spent wearing full PPE. On social media, nurses speak of crippling tiredness after long shifts with sore faces after so many hours in masks, as well as communication barriers with colleagues and patients when wearing full PPE; nurses often cannot hear patients, and patients can struggle too; not being able to see nurses' faces or hear what is said.

Nurses also speak of the difficult ethical and moral judgements that are being taken in hospitals; care homes and the community throughout the world. They tell of experiencing stigma in the wider community, being perceived as a threat to the safety of others and as “disease‐carriers.” As the number of COVID‐19 patients grow, there will be increasingly stringent rules about who can be offered ventilation, with one doctor suggesting “soon many of our own staff would not meet the criteria” (Anon & The Guardian, 2020). Reflecting on the Italian College of Anaesthesia, Analgesia, Resuscitation and Intensive Care (SIAARTI) COVID‐19 guidelines for the criteria that doctors and nurses should follow, the moral philosopher Yascha Mounk reflects: “If you are an overworked nurse battling a novel disease under the most desperate circumstances, and you simply cannot treat everyone, however hard you try, whose life should you save?” (Mounk, 2020). Nurses are likely to experience moral and ethical conflict with the potential for stress and moral distress or moral injury (Bridges et al., 2013; Greenberg, Docherty, Gnanapragasam, & Wessely, 2020; Morley, Ives, Bradbury‐Jones, & Irvine, 2019). These stressors are present across settings in health and social care, and relevant to all members of the nursing team, including care assistants and temporary members of the team drafted in from their studies or from retirement. There is also an emerging narrative of guilt and some of potential shaming among nurses and students who are unable to contribute to direct patient care due to their own high risk and vulnerability to coronavirus.

Nurses and their unions are speaking up about the lack of testing for front‐line staff and the variation in access to PPE. Nationally and internationally it appears there is wide variation in access to PPE and the Royal College of Nursing in the UK and its counterparts across the world have been campaigning for adequate PPE for nurses suggesting the nursing voice has been side‐lined in the relevant debates (Ford, 2020). In many countries, the focus has been on acute and intensive care; however, nurses in the community and mental health and learning disability settings may also have inadequate access to PPE. The UK priorities for PPE distribution and testing are being interpreted as further discrimination against nurses who do not work in acute physical health settings, leading to further anger that some lives appear to matter less. A failure to protect nursing staff adequately is causing anger and frustration, making nurses feel unsafe at work, while they are risking their own health and fearful of transmission to their families. Unless nurses feel well supported by their organisations and governments, that anger may linger after the crisis potentially causing some to leave the profession.

It would be difficult as a nurse not to have strong emotional reactions to the COVID‐19 virus and its impact on one's work (fear, anger, frustration, worries). Such fear and anxieties are normal, as are the intense feelings evoked when nurses feel unable to care for patients as they would have otherwise. Nurses and healthcare or nursing assistants, in acute, community mental health and social care settings are having to make extremely difficult decisions from one moment to the next. They are having to be very creative about new ways of working with very ill patients with mental health needs or learning disabilities or dementia. Legal frameworks to support the continuation of care at times of mental health crises such as (in the UK) potential temporary amendments to the Mental Health Act 1983 and the Coronavirus Act 2020 which enforce isolation; place further strain on therapeutic relationships and the delicate balance between nursing care and restrictive practice. In situations where compliance with social distancing and isolation with COVID‐19 is low on the list of priorities for people in receipt of care, nurses are having to weigh up human rights, safeguarding and infectious disease protocols all of which may potentially conflict. There are some good signs that health systems are recognising how important it is to support healthcare staff. In the UK, NHS staff have been given free access to more than 1,500 specialists, online therapy and group counselling sessions and will receive practical and financial assistance as well as specialist bereavement and psychological support. Volunteers from charities including Hospice UK, the Samaritans and Shout are staffing phone and text helplines. The NHS is also offering free access to support from Apps such as Headspace, UnMind and Big Health for healthcare staff and their families to include guided meditation and tools to battle anxiety and help with sleep problems. This is a good start, but these services rely on the individual seeking help, and this may well not be sufficient. Investment in a range of supportive measures that do not just place the onus on the individual is almost certainly necessary.

## 2 EVIDENCE‐BASED PSYCHOLOGICAL SUPPORT

Supporting nurses practically and psychologically is essential to preserving their health in the short and long term, particularly when occupational stress levels are so high. Ensuring psychological well‐being requires a layered response, with different components at different times, comprising strategies aimed at prevention through to treatment, and strategies/actions at different levels, from organisational and team/ward responses to those aimed at individual self‐care and peer support. Response to the specific unprecedented challenge of COVID‐19 will also need a flexible strategy as needs and requirements are likely to change over the course of the pandemic response. Furthermore, nurses working outside acute hospitals, working autonomously or in dispersed teams across large geographical areas can find accessing support challenging.

**FIGURE 1:**
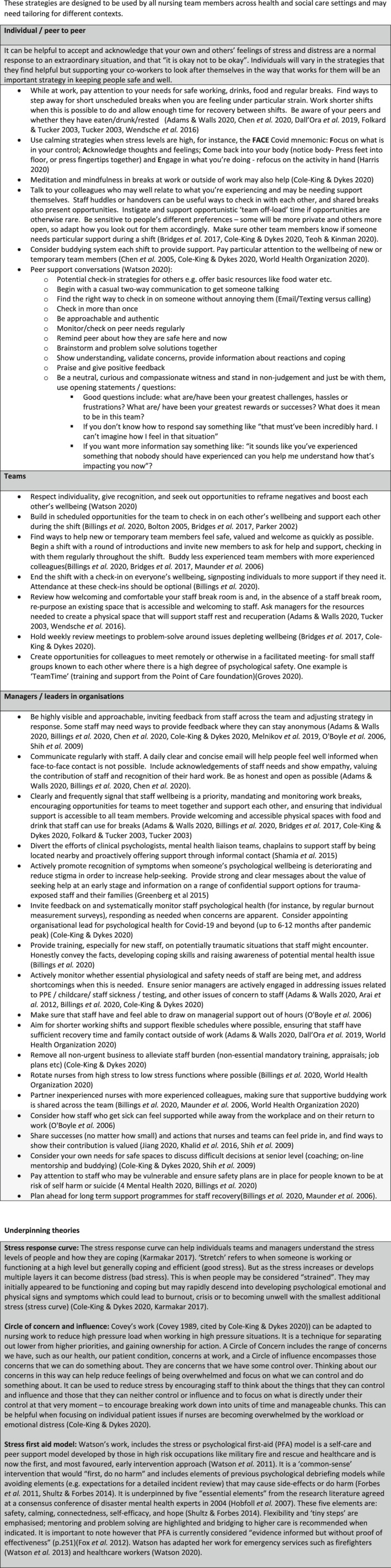
Strategies and interventions to support nurses' psychological well‐being during Covid‐19 crisis. Figure 1 reproduced with permission: Maben, J. Taylor C. and Bridges, J. 2020. Guidance to support nurses’ psychological well‐being during Covid‐19 crisis. University of Surrey and the University of Southampton © Published online 21.4.20. https://www.surrey.ac.uk/sites/default/files/2020-04/guidance-to-support-nurses-psychological-well-being-during-covid-19-crisis.pdf

Having reviewed the literature and gathered intervention resources from a variety of sources, it is evident that there is much to learn from other similar crisis situations such as SARS, MERS and Ebola. The evidence base in this area is considered weak, and most research is observational or has focussed on early interventions after major incidents and once the crisis has passed (Billings et al., 2020). From a nursing perspective, few studies consider nursing outside of hospital walls. In Figure 1, we present strategies and interventions aimed at supporting nurses' psychological well‐being during the COVID‐19 crisis. This guidance is led by best‐available evidence, underpinned by theory (see Figure 1), expert opinion and models used in the military, as well as experiences from other countries and other infectious disease outbreaks (Watson, 2020; Watson, Brymer, & Bonanno, 2011; Watson et al., 2013). Below, we highlight physiological and safety needs; peer support; team support; and the roles and needs of managers and leaders as well as long‐term recovery support needs.

## 3 ADDRESS YOUR OWN PHYSIOLOGICAL AND SAFETY NEEDS

While at work and outside of work, nurses should prioritise their own well‐being as much as possible, paying attention to meeting their essential needs for drinks, food, rest and sleep, and building in rest and comfort breaks (Cole‐King & Dykes, 2020). At times of crisis, human physiological and safety needs come to the forefront—adequate food, shelter, rest, sleep and safety needs for example (Kenrick, Griskevicius, Neuberg, & Schaller, 2010). Recent interviews with medical staff (including nurses) treating COVID‐19 in a hospital in Hunan Province support this (Chen et al., 2020). A detailed psychological intervention package (online course to deal with psychological problems; a psychological assistance hotline; and group interventions) encountered obstacles, as staff were reluctant to participate. Staff reported not needing a psychologist at this time, as they were concerned with more immediate worries including not wanting their families to worry; more rest without interruption; enough protective supplies; and support and training with patients' anxiety and panic. The psychological intervention measures were therefore adjusted to include a place to rest; guaranteed food and daily living supplies; videos of their work to share with families to alleviate concerns; training to manage patient's psychological problems; and access to security staff for un‐cooperative patients. Finally, psychological counsellors regularly visited the rest areas to listen to staff difficulties and stories and provide support accordingly (Chen et al., 2020). In countries still grappling with the threat of the surge from the virus, there are calls from nurses for adequate PPE; hot food; and access to testing. Getting the timing right for psychological support chimes with the wider evidence base that intervening too early with more intense support, for example psychological debriefing, can be harmful (McNally, Bryant, & Ehlers, 2003; Rose, Bisson, Churchill, & Wessely, 2009).

## 4 PEER SUPPORT: LOOK AFTER EACH OTHER

Drawing on the work of Patricia Watson (Watson, 2020; Watson et al., 2011, 2013), we know that akin to the armed forces, healthcare work is team‐driven. Those injured by stress may be the last to recognise it and stigma can be an obstacle to asking for help. Thus, individuals often do not prioritise taking good care of themselves; recognising it may put pressure on colleagues; or they fear letting the team down. However, without looking after self, nurses cannot look after others. Yet health professionals are “wired” to look after others and not self—they are therefore likely to need others (colleagues, friends (peers) and managers) to remind them to think of themselves. For example, last week a colleague spoke of getting a glass of water for a nursing team member who had not had a drink for more than 8 hr. See Figure 1 for strategies and interventions for individual and peer support.

## 5 TEAM SUPPORT: INTERVENTIONS TO SUPPORT TEAM WELL‐BEING

During the pandemic, nurses may be working with people who are not their usual team colleagues. Teams therefore need to support each other and find ways to help new members feel safe, valued and welcome as quickly as possible (see Figure 1). Buddying with more experienced colleagues can help support colleagues who have returned from practice, have been redeployed or are final‐year students who are counted in the numbers or in some countries have been registered early (Maunder et al., 2006). However, buddying needs to be closely monitored, so that the same people are not overburdened. Managerial support and resourcing should be provided so that buddying is not seen as an easy way out for organisations meaning they are not providing adequate psychological or other support.

All members of the team—temporary and longer‐standing—need access to support during and after the shift. In the Creating Learning Environments for Compassionate Care (CLECC) study (Bridges et al., 2018), registered nurses and healthcare assistants identified the benefits of nursing teams engaging in mid‐shift cluster discussions to check in on each other's well‐being. This echoes other findings; that the creation of unmanaged spaces for work‐team members to “take shelter” (Bolton, 2005:134) provides valuable learning and social support for nurses undertaking difficult work with clients (Bolton, 2005; Parker, 2002). Staff in the CLECC study welcomed an opportunity to briefly meet mid‐shift to check in on each other's well‐being (well‐being was the primary focus, unlike safety huddles) and other evidence suggests huddles at the beginning and end of shifts can also help to activate social support for each other (see Figure 1). In noncrisis times, there is evidence that group reflective spaces such as Schwartz Rounds can reduce stress, improve team connectedness and increase compassion for self, colleagues and patients (Maben et al., 2018). However, to maintain psychological safety in Rounds, our study confirmed that staff stories told by panel members should not be too “raw”; staff should have had the opportunity to process and digest a difficult case or experience to protect staff from poor adjustment (Rose et al., 2009). Thus, Schwartz Rounds as previously run (open to all in the organisation and monthly face to face) may not be the “right” solution at the height of the pandemic. The Point of Care Foundation in the UK is therefore adapting their expertise in Schwartz Rounds to offer training in “Team Time.” Team Time has the core features of Schwartz Rounds, but in a virtual format to be run in smaller existing teams (not across the whole organisations); with careful selection by experienced facilitators of “safe” stories and for a shorter time (30 min instead of an hour) with two facilitators present (Groves, 2020). These Team Time sessions are being implemented in clinical practice and in student and other teams in the next few weeks and we plan to use them with our 3rd‐year students at the University of Surrey. Importantly, these adaptations will require timely evaluation.

## 6 ROLES AND NEEDS OF MANAGERS AND LEADERS

A consistent finding from studies of members of the armed forces is that team cohesion horizontally (between colleagues) and vertically (between leaders and their teams) is highly correlated with mental health, with a reported 10‐fold difference in trauma‐related mental health status between troops who perceived themselves as having a good or bad leader (Jones et al., 2012). There is therefore much managers and leaders can do to support nurses in their teams and organisations (see Figure 1 for strategies and interventions). Evidence‐informed guidelines also suggest clear regular and honest communication is key as well as visibility and ensuring access to physiological and safety needs (Billings et al., 2020; Cole‐King & Dykes, 2020). It is also important that senior nurses seek support for themselves, so that they have the capacity to support others and are able to role model good self‐care. Opportunities to process decisions and access to a reflective space are particularly important for senior nurses, where they can think through the difficult decisions they are having to make in response to COVID‐19 challenges. They will need their nursing/ healthcare friends and peers to lean on during the pandemic. Buddying or seeking a respected mentor for confidential peer support is therefore important.

## 7 LONG‐TERM RECOVERY

Evidence tells us it is important not to pathologise what are normal fears and anxieties in such extraordinary and frightening situations and stress zones, and that needs change over time (Billings et al., 2020). Most individuals exposed to highly challenging or traumatic events exhibit resilience and do not suffer any long‐term negative psychological effects (Rubin, Brewin, Greenberg, Simpson, & Wessely, 2005). However, inevitably some will suffer distress; in most cases, these symptoms resolve without the need for any formal interventions (Greenberg, Wessely, & Wykes, 2015).There is evidence of staff experiencing post‐traumatic stress disorder (PTSD) after previous infectious disease outbreaks for example Ebola (Greenberg et al., 2015)—but that does not mean all staff will experience poor mental health outcomes. However, it will be important to understand the psychological needs of the nursing workforce following the height of the pandemic, so that individually, in teams and across organisations as well as across nations, we can learn together and make sure nurses have access to adequate support in the subsequent recovery phase to avoid a generation of nurses with poor psychological health. Post‐pandemic or in the tail of the acute phase staff may be “running on empty” some may have unresolved grief, if staff colleagues have died or family members (and there has been no time to grieve), and some may have burnout or possibly retrospective guilt (Cole‐King & Dykes, 2020; Greenberg et al., 2015). It is important that interventions do no harm and are tailored to individual need. Attendance at talking therapies and reflective groups should be optional. If conducted too early or inappropriately, detailed processing could disrupt an individual's normal coping mechanism and psychological debriefing is now undertaken with great care. A body of controlled research has raised serious questions regarding the timing of the beneficial effects of debriefing, especially as a one‐off event, in terms of long‐term recovery. Importantly, for a minority of recipients, psychological debriefing may actually result in worse adjustment (McNally et al., 2003; Rose et al., 2009).

Access to treatment for those staff who require management of established trauma‐related mental health problems such as PTSD (e.g. trauma‐focused cognitive behavioural therapy and Eye Movement Desensitisation and Reprocessing which are known to be effective [NICE 2005 in Greenberg et al., 2015]) may be required. Importantly, extra funding may be needed for this, and it will be important that there is equity of access and that this is available to nurses as well as other members of the healthcare team.

## 8 CONCLUSION

Clearly, to “get through” this unprecedented situation, some resilience is needed but nurses need their employers, their teams, the profession and the public to support them with action and resources. #ClappingForTheCarers and publicly applauding front‐line staff daily/ weekly throughout Europe is helping to lift spirits, and some nurses report feeling moved at the collective acknowledgement of gratitude and donation of gifts such as food and other gifts. There are also reports of teams pulling together in cooperative effort and great camaraderie in emergency departments and intensive care units to name but a few. But this is not enough, nurses also need to feel *their* needs are cared for and that they are safe with adequate PPE equipment in *all* settings where health and social care are being delivered. They need access to rest breaks, good peer and team support and leaders that will continue to care for them well after the pandemic is over. As researchers who have studied nurse well‐being for decades, it is gratifying to see the increased focus on healthcare staff well‐being, yet sad that it takes a pandemic to recognise its critical importance. Yes, staff will need “resilience” but resilience must never be seen as an individual responsibility, it is a collective and organisational responsibility. Evidence from the military suggests the resilience of the team appears to be “more related to the bonds between team members than the psychological make‐up or coping styles of any individual” (Greenberg et al., 2015). The word “resilience” (and resilience training) is now rightly contested as staff can feel it is another stick to beat them with; if staff are stressed and struggling psychologically (through lack of resources or ethical and emotional challenges as in COVID‐19), nurses can feel it is their “fault” because they have not implemented the training adequately or been “resilient enough.” This is neither true not acceptable.

Let this be an opportunity to fully recognise the inherent stresses and emotional strain that nurses bear on behalf of society and ensure support, not only through this crisis but after it is all over. When health care is back to “normal,” ongoing support for nurses' well‐being will remain critically important. While COVID‐19 places particularly high stress on nursing, there is very little in the guidance presented here that was not relevant to staff well‐being “pre‐COVID” and when the pandemic is over, we look forward to the guidelines being used to establish better support for nurses and nursing into the future.
